# Ultrasound responsive carbon monoxide releasing micelle

**DOI:** 10.1016/j.ultsonch.2020.105427

**Published:** 2020-12-26

**Authors:** Osamah Alghazwat, Somayeh Talebzadeh, Jeremiah Oyer, Alicja Copik, Yi Liao

**Affiliations:** a Florida Institute of Technology, Melbourne, FL, USA; b University of Central Florida, Orlando, FL, USA

**Keywords:** Carbon monoxide releasing material, Ultrasound, Micelle, Drug delivery

## Abstract

Carbon monoxide (CO), an endogenously produced gasotransmitter, has shown various therapeutic effects in previous studies. In this work, we developed an ultrasound responsive micelle for localized CO delivery. The micelle is composed of a pluronic shell and a core of a CO releasing molecule, CORM-2. The mechanism is based on the ultrasound response of pluronics, and the reaction between CORM-2 and certain biomolecules, e.g. cysteine. The latter allows CO release without significantly breaking the micelles. In a 3.5 mM cysteine solution, the micelles released low level of CO, indicating effective encapsulation of CORM-2. Treatment with a low intensity, non-focused ultrasound led to four times as much CO as the sample without ultrasonication, which is close to that of unencapsulated CORM-2. Significantly reduced proliferation of prostate cancer cells (PC-3) was observed 24 h after the PC-3 cells were treated with the CORM-2 micelles followed by ultrasound activation.

## Highlights

• Development of the first ultrasound responsive carbon monoxide releasing material.
• Activation by relatively low intensity of ultrasound.
• Material composed of FDA approved polymer and a well-accepted carbon monoxide releasing molecule.

## 1 Introduction

Carbon monoxide (CO) is produced naturally during heme catabolism. As a gasotransmitter, CO plays important roles in many physiological functions in the mammalian body. [1] Studies have shown that CO has beneficial effects including anti-inflammatory, anti-apoptotic, anti-coagulative, anti-hypertensive, cell protective effects etc. [2], [3], [4], [5], [6], [7], [8], [9], [10] In the preclinical and animal studies, CO was delivered by inhalation or using CO releasing molecules (CO-RMs). Inhaled, small-quantities of supplemental CO gas has been demonstrated in pre-clinical disease models to have therapeutic effects including reducing inflammatory and cardiovascular disorders etc. [11] CO-RMs are a group of compounds capable of releasing controlled quantities of CO in cellular systems. [12], [13], [14], [15], (a), (b) The majority of CO-RMs studied are carbonyls of transition metals including both essential trace elements (manganese, iron, cobalt) and non-physiological metals (ruthenium, tungsten, rhenium). [1], [4], [5], [6], [7], [8], [9], [10] Some nonmetallic CO-RMs have also been developed in recent years. [14], [15], [16], [17], [18], [19], [20], [21], [22] CO-RMs allows convenient administration of a certain dose of CO. In addition, the potential to control the release of CO to a specific target is the major advantage of CO-RMs as a therapeutic agent. For this purpose, CO releasing molecules and materials [23] that response to different stimuli including light, magnetic field, enzyme, and reactive oxygen species (ROS) have been developed in recent years. [15], [16], [17], [18], [19], [20], [21], [22], [23], [24], [25], [26], [27], [28], [29]

Ultrasound (US) imaging is one of the most widely used diagnostic methods. US has also been used in tissue ablation, stone crushing, and transdermal drug delivery etc. [30] In the past decade, US mediated drug delivery has been intensively studied. [31], [32], [33], [34], [35], [36], [37], [38], [39], [40], [41], [42] US either interrupts cell membrane, which increases cellular uptake of drugs, or interacts with the nano/micro drug carrier and releases the drug encapsulated. Given the deep penetration of US and well-developed US technology, US is an attractive approach for spatial and temporal control of drug delivery. Recently, we reported the first US responsive CO releasing material in a *chemrxiv* preprint. [43] Herein we report the detailed study as well as much improved results of this work. In addition, the effect of the material on prostate cancer cell was studied.

## 2 Results and discussion

**Fig. 1:**
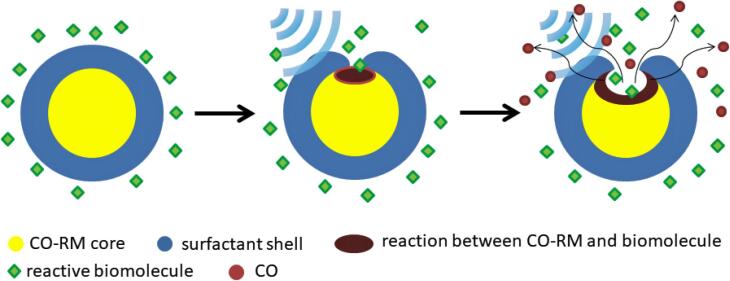
Illustration of the US mediated CO release based on a reaction between a CO-RM and a common biomolecule.

Many CO-RMs react with common species in biological systems, e.g. water, cysteine, etc. to release CO. They can be considered as prodrugs of CO. When they are encapsulated in nanocarriers, the CO releasing rate will be significantly reduced. If US can induce leakage on the shell of the nanocarrier, not only the encapsulated CO-RM can move out of the nanocarrier to react with the biomolecules, but also the reactive biomolecules can go inside to react with the CO-RM and release CO. (Fig. 1) The latter mechanism is important since the compounds encapsulated in nanocarriers (in this case the CO-RM) are often hydrophobic and thus difficult to escape from the nanocarrier. Since CO is a gas, it can be quickly released from inside of the nanocarrier. Therefore, the shell of the nanocarrier does not have to be significantly destructed to cause significant drug release, which means a relatively low intensity of US may be used.

CORM-2 [Ru_2_Cl_4_(CO)_6_] is one of the most extensively studied CO-RM. It has shown various therapeutic effects in many animal tests. [44] It was thought that CORM-2 quickly releases CO via hydrolysis. However, recent studies by Poole and coworkers showed that CO release from CORM-2 was actually very slow in water and very quick in the presence of active sulfur compounds including cysteine and sodium dithionite. [45] The latter is commonly used in the myoglobin test for measuring the concentration of CO. Since cysteine, glutathione, and proteins with free cysteine residues are abundant inside cells [46], CORM-2 is a promising candidate for preparation of US responsive CO releasing material. Its hydrophobicity and low reactivity with water allow encapsulation with common nano-precipitation or solvent evaporation methods. Pluronics are FDA approved drug delivery polymers, which form micelles in water. Previous studies have shown that pluronic micelles responded to US and released drugs encapsulated. [47], [48], [40], [41], [42] Therefore, we used pluronics as the surfactants for the preparation of the CORM-2 micelles.

Pluronic micelles containing CORM-2 were prepared by adding an acetone solution of CORM-2 and pluronic F-127 dropwise to a stirred aqueous solution of the pluronic. The weight ratio between CORM-2 and the total amount of pluronic F-127 was 1:5. The solution was stirred in an open vial for 40 min to evaporate most of the acetone, and then lyophilized to yield a white powder. Details are given in Experimental section. This is an improved procedure comparing to the one in the preprint [43] reported earlier. This procedure allows the CORM-2 to be effectively encapsulated by pluronic and largely reduces the amount of CO released in the absence of US. It should be noted that the CORM-2 used has a formula of Ru_2_Cl_4_(CO)_6_·1/4 H_2_O. We found that the water in the material greatly increased its solubility in acetone.

**Fig. 2:**
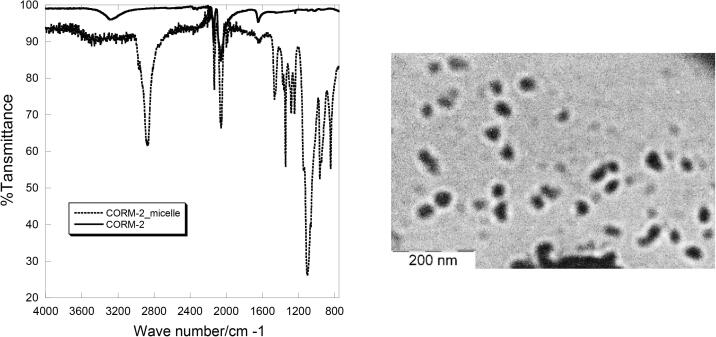
IR spectrum (left) and TEM image (right) of the CORM-2/pluronic micelles.

The white powder obtained after lyophilization was a little hydroscopic and turned sticky after some days. Therefore, it was stored in a vial filled with nitrogen in refrigerator. In this case, no change of appearance was observed after a month. Infrared spectroscopy showed strong CO stretching peaks at 2064 cm^−1^ indicating that CORM-2 was not decomposed during the preparation. The size of the micelles was studied by dynamic light scattering (DLS) and Transmission electron microscopy (TEM). (Fig. 2) DLS showed a mean size of 109 ± 16 nm with a PI of 0.14. TEM showed dark particles with diameters of ~ 50 nm, which is much smaller than that from DLS. Since the heavy metallic Ru(II) ion is much more sensitive to TEM than the pluronic shell, the dark particle observed by TEM is the CORM-2 core.

**Fig. 3:**
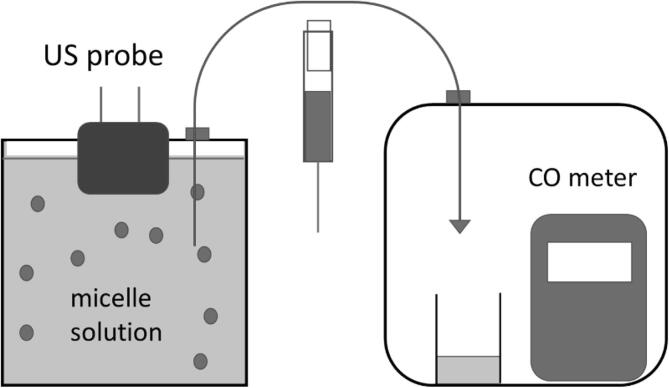
Setup for ultrasonicating the micelle solution and measuring the CO release.

The CORM-2/pluronic micelles were resuspended in water and the CO release from the micelles was studied. CO release from CO-RMs is commonly measured by myoglobin test and/or head space method using a CO meter. As mentioned above, the sodium dithionite used in myoglobin test can react with CORM-2 and release CO. Therefore, we used the head space method with a setup commonly used in CO-RM research. (Fig. 3) Basically, a small amount of the CO-RM solution in an open vial is sealed in a bigger container with a CO meter. The CO released from the CO-RM solution can be calculated using the following equation [49]:

(1) $$N_{\mathrm{CO}}=\frac{pV_{g}}{\mathrm{RT}}+cV_{l}=p\left(\frac{V_{g}}{\mathrm{RT}}+\frac{V_{l}}{k}\right)$$

[where *p* is partial pressure of CO (CO meter readings); *V_g_* is volume of the gas phase; *V_l_* is liquid phase; *R* is 0.08205 L·atm·mol^−1^·K^−1^; *T* is Temperature; *c* is CO concentration in the liquid phase; *k* is Henry’s law constant of CO in water (1052.63 L·atm·mol^−1^ at 25 °C)].

As expected, no CO was detected when the aqueous suspension of CORM-2/pluronic micelles was tested. A concentrated solution of cysteine was then added to a CORM-2/pluronic micelle suspension. The final concentration of cysteine and CORM-2 was 3.5 and 0.35 mM respectively. In previous works, the cysteine concentration used for CO release from CORM-2 and its derivatives was 1–10 mM [45], [40], [50], which was close to the concentration used in this study. Three mL of the solution was transferred to the setup of CO measurement (Fig. 3 right). After 15 min, the CO released to the setup was 1.5 ppm.

**Fig. 4:**
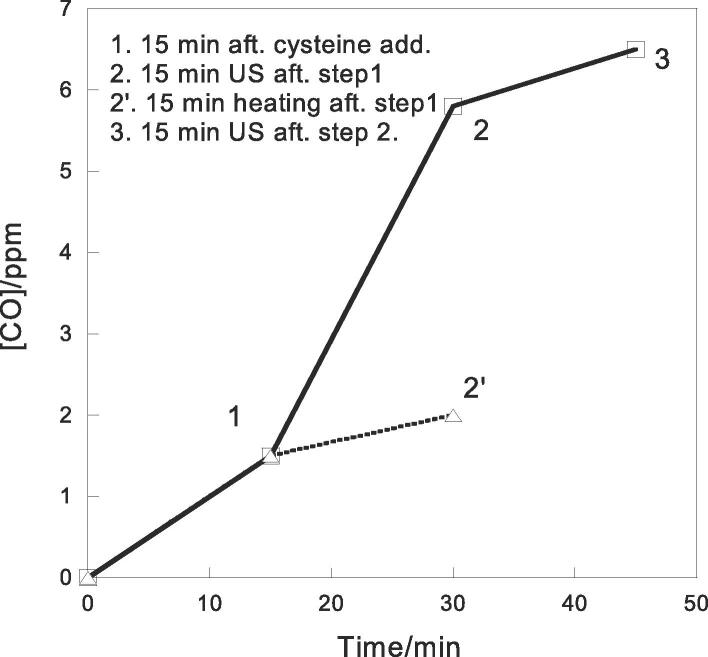
[CO] detected after addition of cysteine, ultrasonication, and heating.

To test whether US can assist the release of CO, the solution of the CORM-2 micelles and cysteine was ultrasonicated. A commercial therapeutic US equipment was used to apply non-focused US to the micelle solution. Therapeutic US equipment has been used before for *in vitro* evaluation of US responsive materials. [51] The micelle solution was sealed in a container with the US probe ~ 3 mm below the surface. (Fig. 3 left) The frequency was 1 MHz; intensity was 2.5 W/cm^2^; and the duty cycle was 1:1. After the suspension was ultrasonicated for 15 min, 3 mL of the solution was taken with a syringe from a small hole sealed with rubber, and then transferred to the head-space setup with a CO meter. A steady reading of 6 ppm was observed, which was 4 times higher than the CO release without US. (Fig. 4) The experiment was repeated five times using different batches of the micelles. Increase of CO release after ultrasonication was observed every time. The average of CO released after ultrasonication was 5.8 ± 0.5 ppm. Ultrasonication for additional 15 min further increased CO concentration to 6.5 ± 0.6 ppm. Prolonged ultrasonication did not lead to observable increase of CO release.

We noticed that ultrasonication increased the temperature from room temperature (~25 °C) to ~ 31 °C during the 15 min period. In fact, this is the reason that we used 1:1 duty cycle instead of continuous mode since the continuous mode raised the temperature to nearly 40 °C. To confirm that the CO release increase was not due to a thermal effect, the CORM-2/pluronic suspension with cysteine was kept at room temperature for 15 min, and then put in a heating bath at 30 °C for another 15 min. After transfer 3 mL of the heated solution to the measurement setup, the CO meter showed a reading of 2 ppm, which is higher than that of the unheated sample, but much lower than the ultrasonicated one (Fig. 4). Increasing temperature to 40 °C did not lead to more CO release.

The molar number of CO calculated from equation (1) was divided by the molar number of CORM-2 to calculate the percent yield of CO. We assume that the percent yield of CO is the percent of the CORM-2 reacted since it indicates the average reactivity of the CORM-2 in the micelles. Calculation showed that before sonication, 2.5% of CORM-2 released CO in the presence of 3.5 mM cysteine. After sonication for 15 min, 9.5% of CORM-2 released CO. The percentage of reacted CORM-2 increased to 10.6% after additional 15 min sonication. The amount CORM-2 reacted (~10%) in cysteine solution (3.5 mM) is comparable with the previously reported values for CORM-2 and its derivatives especially those with polymers. [45], [49], [50] Previous work showed that vasodilatory activity level of 150–300 μM CORM-2 was comparable to a 10 μM CO solution on isolated rat afferent arterioles [52], which indicates that therapeutic effects can be generated from the CO released from a few percentage of CORM-2.

It is important to compare the reactivity of US activated CORM-2 micelle with unencapsulated CORM-2. Therefore, a control experiment was conducted using unencapsulated CORM-2. CORM-2 was dissolved in DMSO. The solution was diluted with water and then transferred to the setup for CO measurement. A stock solution of cysteine was added to the CORM-2 solution via a rubber seal using a syringe. The final concentrations of COMR-2 and cysteine were 0.34 mM and 3.4 mM respectively, which are about the same as that of the micelle tests. After 30 min, the concentration of CO gas in the setup was 6 ppm. The result showed that ultrasonication allows the CORM-2 to react like unencapsulated CORM-2.

**Fig. 5:**
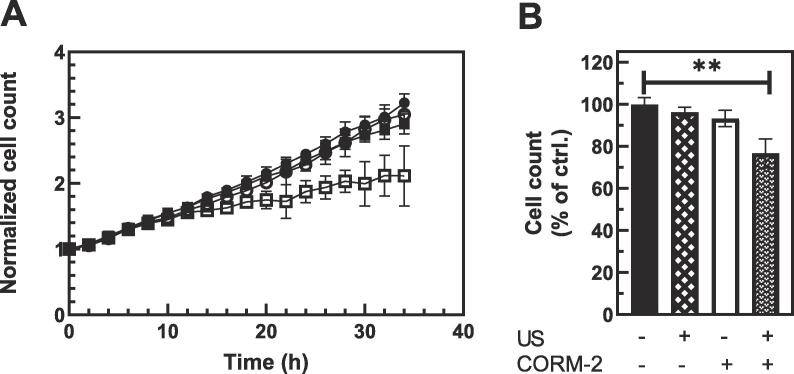
Biological effects of US activated CORM-2 micelles. [A. PC-3 prostate cancer cells were incubated overnight with CORM-2 micelles (CORM-2 -open symbols) or media alone (closed symbols) and then were either treated (□,■) or not (○,●) with 1 MHz US for 3 × 5 min. Each data point represents average of 3 wells with 4 images averaged per well.] [B. Cell content in treatment wells was plotted as a percent of average cell count in control wells at 24 h time point.]

Next, the effects of CORM-2 micelle on prostate cancer cells (PC-3) were tested. Prostate cancer cells were either left untreated or treated overnight with 40 µM of CORM-2 micelles and then exposed to three 5 min treatment of US. The equipment and parameters of ultrasonication were the same as that of the experiments described above. The US was applied from the bottom of BioFlex® culture plates through a layer of US gel. The growth of cells was then monitored over period of 34 h using live-imaging system. As shown in Fig. 5, treatment with either US or CORM-2 micelles only did not cause significant change in proliferation of PC-3 cells while treatment of cells with both CORM-2 micelles and US lead to significant decrease in cell number in the treated wells. At 24 h time-point, cell counts in the wells treated with both US and micelles were decreased to 76% (p = 0.006) of the cell content in untreated control wells and to 66% by 34 h. This is consistent with cytotoxic effects of CO on prostate cancer cells reported before. [53], [54] Especially, Yan et al. reported that applying 40 µM of CORM-2 to PC-3 cells resulted in a cell viability ~ 70% of the untreated cells. [54] The effect is close to that of the CORM-2 micelles after US. Details of the experiment are described in Experimental Section.

In summary, we found that CORM-2 was effectively encapsulated by pluronic, which resulted in low level of CO release in the presence of cysteine. Non-focused, low intensity US from a common therapeutic equipment is strong enough to allow the CORM-2 micelles to react the same as unencapsulated CORM-2 and release about 4 times as much CO as the sample without sonication. Treatment of prostate cancer cells with the CORM-2 micelles followed by US activation significantly reduced the proliferation of the cancer cell. Using pluronic, which is an FDA approved drug delivery polymer, and CORM-2, which has been studied in many animal tests, as the components is a major advantage and could allow animal studies in near future.

## 3 Experimental section

### 3.1 General methods

Unless otherwise noted, reagents and solvents were commercially available and used as received without any further purification. CORM-2 with a formula of Ru_2_Cl_4_(CO)_6_·1/4 H_2_O was purchased from Tocris. Pluronic F-127 was purchased from Sigma Aldrich. Ultrasonication was conducted using a Physio Sound UT ultrasound physical therapy machine. Dynamic light scattering (DLS) measurements were carried out on a SZ-100 Nanopartica Series Instrument manufactured by HORIBA. TEM image was taken with a Zeiss EM900 Transmission Electron Microscope. Partial pressure of CO was measured using a Honeywell BW Solo Gas Detector.

### 3.2 Preparation of CORM-2/pluronic micelles

Pluronic F-127 (35 mg) was dissolved in 2.5 mL of deionized water. To this solution, was added a solution of 10 mg CORM-2 and 15 mg of Pluronic F-127 in 1 mL of anhydrous acetone. The solution turned cloudy after addition. The mixture was stirred for 45 min in an open vial to evaporate most of acetone. and then was lyophilized for 4.5 h, which yielded a white powder. The product was kept in a closed vial filled with some nitrogen in a refrigerator.

### 3.3 Evaluation of the CO release from the CORM-2/pluronic micelles

The CORM-2/pluronic micelles (60 mg) were dissolved in 55 mL of deionized water and transferred to a 60 mL container with the US probe on the top of the container through a hole. The small gap between the probe and the edge of the hole was sealed with vacuum grease. After the container was closed, 2 mL of stock solutions of cysteine was added via a hole sealed with rubber using a syringe. At this time, the probe was about 3 mm below the surface of the solution. The final concentrations of CORM-2 in the micelles and cysteine were 0.35 and 3.5 mM respectively. After 15-min reaction, 3 mL of the mixture was taken by a syringe and transferred to the head-space setup (Fig. 3) to measure the CO released from the micelles. US was then applied to the remaining mixture. The frequency was 1 MHz; intensity was 2.5 W/cm^2^; and the duty cycle was 1:1. After 15 min of ultrasonication, 3 mL of the mixture was transferred to the head-space setup for CO measurement. Another 15 min of ultrasonication was applied to the remaining mixture and then the CO released was measured as above.

### 3.4 Evaluation of biological effects of US activated CORM-2/pluronic micelles

PC-3 prostate cancer cells were maintained between 30 and 90% confluency in RPMI 1640 media supplemented with 10% FBS and 1% antibiotic/antimycotic (PC-3 growth media) in 37 °C, 5% CO_2_ environment. For CORM-2 micelle treatment, PC-3 cells were lifted using 0.25% Typsin for 6 min at 37 °C, and re-seeded at 200,000 cells per well in 6-well plates in PC-3 growth media. Cells were allowed to attach for 24 h in 37 °C, 5% CO_2_ environment. PC-3 cells were then incubated for 18 h with 40 µM CORM-2 micelle or media alone and then were either treated or not with 1 MHz US for 3 × 5 min at room temperature. The US was applied from the bottom of BioFlex® culture plates through a layer of US gel. PC-3 cells were detached with 0.25% Trypsin for 6 min at 37 °C, washed once to remove trypsin and 10,000 cells/well were plated in a 96-well plate. Starting 1 h after seeding, plate was imaged every two hours for 34 h using IncuCyte S3 system (Sartorius) with 4 images recorded at 20x magnification per each well and each condition run in triplicate wells. Phase contrast was used to quantify cell amounts in each image. Cell counts were normalized to the starting cell amount in each well. Unpaired *t*-test was used to determine significance.

## CRediT authorship contribution statement

**Osamah Alghazwat:** Investigation, Validation. **Somayeh Talebzadeh:** Investigation, Validation. **Jeremiah Oyer:** Investigation, Validation. **Alicja Copik:** Supervision. **Yi Liao:** Conceptualization, Supervision.

## Declaration of Competing Interest

The authors declare that they have no known competing financial interests or personal relationships that could have appeared to influence the work reported in this paper.
